# COVID-19 Detection Mechanism in Vehicles Using a Deep Extreme Machine Learning Approach

**DOI:** 10.3390/diagnostics13020270

**Published:** 2023-01-11

**Authors:** Areej Fatima, Tariq Shahzad, Sagheer Abbas, Abdur Rehman, Yousaf Saeed, Meshal Alharbi, Muhammad Adnan Khan, Khmaies Ouahada

**Affiliations:** 1 Department of Computer Science, Lahore Garrison University, Lahore 54000, Pakistan; 2 Department of Electrical and Computer Engineering, COMSATS University Islamabad, Sahiwal Campus, Sahiwal 57000, Pakistan; 3 School of Computer Science, National College of Business Administration and Economics, Lahore 54000, Pakistan; 4 Department of Information Technology, University of Haripur, Haripur 22620, Pakistan; 5 Department of Computer Science, College of Computer Engineering and Sciences, Prince Sattam Bin Abdulaziz University, Alkharj 11942, Saudi Arabia; 6 Department of Software, Faculty of Artificial intelligence and Software, Gachon University, Seongnam 13120, Republic of Korea; 7 Department of Electrical and Electronic Engineering Science, University of Johannesburg, Auckland Park, P.O. Box 524, Johannesburg 2006, South Africa

**Keywords:** coronavirus, DELM, WHO, COVID-19, diagnosis, healthcare

## Abstract

COVID-19 is a rapidly spreading pandemic, and early detection is important to halting the spread of infection. Recently, the outbreak of this virus has severely affected people around the world with increasing death rates. The increased death rates are because of its spreading nature among people, mainly through physical interactions. Therefore, it is very important to control the spreading of the virus and detect people’s symptoms during the initial stages so proper preventive measures can be taken in good time. In response to COVID-19, revolutionary automation such as deep learning, machine learning, image processing, and medical images such as chest radiography (CXR) and computed tomography (CT) have been developed in this environment. Currently, the coronavirus is identified via an RT-PCR test. Alternative solutions are required due to the lengthy moratorium period and the large number of false-negative estimations. To prevent the spreading of the virus, we propose the Vehicle-based COVID-19 Detection System to reveal the related symptoms of a person in the vehicles. Moreover, deep extreme machine learning is applied. The proposed system uses headaches, flu, fever, cough, chest pain, shortness of breath, tiredness, nasal congestion, diarrhea, breathing difficulty, and pneumonia. The symptoms are considered parameters to reveal the presence of COVID-19 in a person. Our proposed approach in Vehicles will make it easier for governments to perform COVID-19 tests timely in cities. Due to the ambiguous nature of symptoms in humans, we utilize fuzzy modeling for simulation. The suggested COVID-19 detection model achieved an accuracy of more than 90%.

## 1. Introduction

Coronavirus disease (COVID-19) is an infectious virus that the World Health Organization (WHO) declared a pandemic on 11 March 2020, indicating the severity of its global spread [1]. The announcement of the disease outbreak also highlighted the growing concern about COVID-19’s unprecedented scale of transmission and intensity. Its scope distinguishes it as a global health emergency that has spread all over the world. Many countries’ legislative bodies have imposed restrictions, travel restrictions, social distancing, and growing sanitation knowledge. Nonetheless, the infection spreads quite quickly. Many COVID-19-infected patients developed mild to severe lung infections, while others developed severe pneumonia. It is expected that older people with these diseases, as well as those with stubborn and incurable lung and kidney problems, will be more prone to becoming ill and developing additional complications in their bodies that can be harmful.

At the end of December 2019, several people in Wuhan with pathological pneumonia were epidemiologically linked to the Huanan local marketplace. Prior to the epidemic, a variety of animals, including birds and rabbits, were marketed. Next-generation sequencing has identified a new coronavirus [2,3,4]. Some patients have high fevers and diarrhea, and X-rays of the lungs show irregular lung lesions [4,5].

To track the virus’s rapid and widespread coverage, preventative measures were taken to safeguard affected areas. These measures included closing territories without further notice, suspending government programs and schools, and limiting domestic and international travel, among others. The goal was to reduce the likelihood of physical contact between individuals, so as to not spread the new virus. China and other nations suffered enormous economic losses as a result of the lockdown. Because this virus is novel, its severity is unknown, despite its highly contagious behavior and longer incubation period compared with other viruses discovered to date. The prohibition could be lifted prematurely, the outbreak could not be entirely subsidized, and extended controls will result in greater economic loss. It is still unknown how long the virus will remain endemic, and we know very little about its morphology, so time is extremely volatile.

The recent pandemic of COVID-19 is attracting the attention of a number of scientists who are seeking support and ways to respond. Rao et al. [6] suggested a framework for the identification of COVID-19 victims via smartphone. Yan et al. [7] established a forecasting framework to assess patients at high risk at an early stage without transmitting them from moderate to severely sick. Several research articles on the forecast of the coronavirus pandemic have been presented in recent times [8]. The researchers concentrated on developing a new framework focused on artificial intelligence technologies that combine machine learning algorithms and various data modalities [9]. The revised method for the optimized neuro-fuzzy inference system (ANFIS) is proposed in this paper [10]. The Regression framework has been built to estimate the rapid growth in COVID-19 dependent on the overall percentage of participants indicated from countries other than China [11]. Experts produced predictions by ten common machine learning and predictive environmental area frameworks to observe large-scale weather variations. [12].

Because COVID-19 attacks our lungs, we may use X-rays to assess the severity of an infected patient’s lungs as well as other relevant symptoms. Health consultants frequently examine a patient’s symptoms in order to diagnose the disease. Brandi et al. [13] showed that the course of COVID-19 ARDS might be variable and unexpected, affecting not just the lungs but also other organs and systems. Because of this, it is crucial for radiologists to be well versed in these extra-pulmonary problems and to guide treatment decisions by means of dependable and frequent monitoring. Balacchi et al. [14] stated that there was no change in the incidence of CT patterns between the first and second waves, validating the RSNA consensus and the most common radiological COVID-19 characteristics. It is important to keep an open mind about the aged population while considering the prevalence of atypical COVID-19 traits. Brandi et al. [15] stated that, even while fungal co-infections and superinfections are widespread, it was shown that Gram-negative bacteria are the most common co-pathogens in this subset of individuals. Radiologists and physicians still struggle with the difficulty of separating primary viral lung infections from subsequent bacterial and fungus infections. In the surveillance of critically sick patients with COVID-19, imaging plays a crucial role in recognizing any suspicious CT imaging findings that may recommend further laboratory tests and appropriate antibiotic medication. These symptoms may be substituted for specific screening methods for the COVID-19 test by all health professionals capable of analyzing the patient’s specific symptoms. As a result, it is difficult for medical professionals to diagnose suspicious cases based on their symptoms, and it also takes longer when the entire world is in peril. Therefore, receiving aid is more important than living a normal life. Consequently, it is vital to develop an automated simulation model to save money and resources for healthcare professionals to detect COVID-19 based on a valid dataset.

Therefore, COVID-19 identification is essential for specific human and social health influences, with the exception of attributes with the weakest evidence. In this scenario, however, the most accurate predictions are required. This is a complex algorithmic challenge in machine learning [16,17]. The empirical research provides three methods for expanding the framework for forecasting a small number of training datasets [18,19]. One strategy is to expand the collection of training data by including more data that is easily available [20,21]. Another method comprises a pattern with cumulative predictive results, and one of the minimum error methodologies is selected to incorporate the predictive results [22]. The other nominees’ outcomes will be ignored. The third strategy is to concentrate on a single forecasting process, which can have multiple modifiable variables [23].

Many researchers have developed a COVID-19 forecasting framework based on deep learning techniques in recent years. Spagnoli et al. [24] pointed out that the radiomic features could be extracted with the help of semi-automatic segmentation tools, leading to the development of forecasting ML models with effectiveness that fell short of that obtained using clinical variables but was superior to that of models based on radiological observations. Shuai et al. [25] employed computed tomography imaging methods to scan COVID-19 patients with an 89.5% accuracy along with a specificity and sensitivity of 88% and 87%, respectively. Linda et al. [26] introduced an 83.5% precision Deep Convolutional Neural Network named COVID-Net to identify COVID-19 instances through chest X-ray scans. Ayrton [27] employed a limited data collection of 339 frames to train and test employing deep transfer learning methods based on ResNet50 and recorded a 96.2% test accuracy. This study developed the VCDS approach (Vehicle-based COVID-19 Detection System) enabled by Deep Extreme Learning Machine (DELM). This paper suggests an adaptive machine-learning method for COVID-19 prediction. More than 98% of the identification accuracy was achieved in the proposed study.

This study aims to detect severely and slightly infected COVID-19 patients on mobile vehicles using a rapid and cost-effective approach and to classify infection risk. Toward this end, a three-step procedure was utilized:

- To begin, it is important to ascertain the elements that impact the prognosis of the condition, such as the routine blood values and demographic data, that are known to be present at the time of admission;
- Second, the identified characteristics are fed into a DELM model in order to identify patients that are either mildly or severely infected with COVID-19;
- Third, evaluate the performance of the different supervised machine learning models using a variety of different assessment metrics such as accuracy, positive predictive value, negative predictive value, specificity, sensitivity, and F-measure.

The remainder of this article is presented as follows. Section 2 introduces the framework for performing a complete COVID-19 identification. Next, the results of the DELM method are addressed in Section 3. Finally, Section 4 addresses the research conclusions.

## 2. Materials and Methods

### 2.1. System Model

The rapid spread of this disease is well documented only in the early stages of the outbreak, so little evidence is available to support theories and assist in its assessment. A DELM methodology utilizing a small dataset that is required in the light of the three key objectives and the accepted detection system needs to be more effectual than its predecessors (with the highest reliability). Although an efficient method requires optimal performance, it also allows for the use of other reasonable regression time intervals. Our suggested solution helps to improve optimum prediction accuracy under the constraints of minimal information and data accessibility. The key advantages of this research are described as follows:

- Minimizing COVID-19 virus spreading timely;
- Controlling physical contact of Vehicle health consultants (VHC) with the people;
- Mobility in different geographic areas: urban, rural, and borders;
- Achieves emergency planning and positioning of emergency vehicles;
- Facilitates the detection of oncoming diseases based on the appropriate dataset.

The development of a COVID-19 early detection system in humans is extremely important. Nonetheless, precise prediction is a difficult and time-consuming task. This article presents a method for effective COVID-19 system prediction based on DELM. The proposed DELM strategy is divided into three levels: the first is the information-gathering phase; the second is the pre-processing technique; and the third is the evaluation phase. The data collecting layer is used to collect data for analysis. Fundamental information assessment techniques are used during the pre-processing phase to remove anomalies in the data. Finally, during the evaluation phase, the system’s prediction and performance are assessed. The proposed DELM framework is being investigated in order to improve the Vehicle-based COVID-19 Detection System (VCDS).

**Figure 1:**
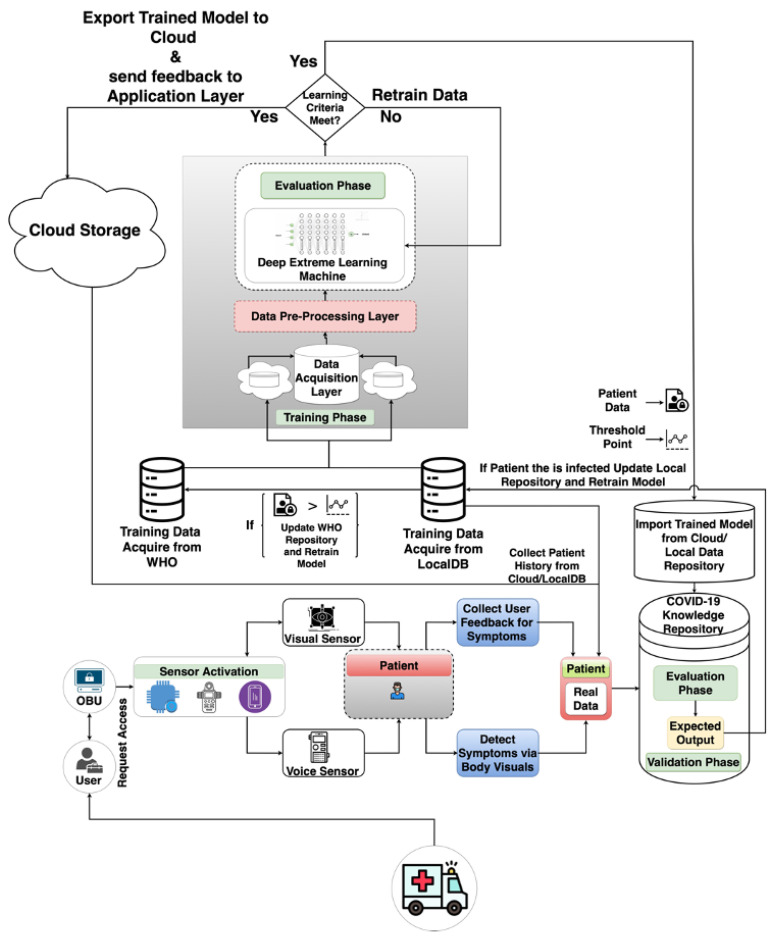
Vehicle-based Approaches to detect individuals with COVID-19 symptoms.

The steps on how VCDS helps to detect COVID-19 are as follows:

- Firstly, the suspected COVID-19 patient is permitted in the Vehicle for testing through VCDS (Vehicle-based COVID-19 Detection System);
- The health consultants activate the VCDS to test the patient for infection. The activation directs the control signal to the OBU (On-Board Unit) of the Vehicle. The onboard unit in VCDS is the central controller responsible for carrying out the entire testing mechanism;
- The onboard unit activates the sensory module that triggers voice and visual sensors. The voice sensor enquires about the symptoms of the patient via Natural Language Processing (NLP). Also, the patient is monitored visually through a camera using machine learning techniques to predict the symptoms. The symptoms are predicted in the COVID-19 knowledge repository;
- In the knowledge repository, the disease will be predicted in the validation phase. And in the validation phase, the trained model and real patient data will be evaluated to predict the disease. The evaluation process will be performed by importing a trained model from a cloud or local repository in which the dataset had been trained through a deep extreme learning machine (DELM). Additionally, we used various statistical methods to quantify the contribution using the DELM algorithm’s corresponding protocols;
- Actual data from the WHO repository or local database are collected. Upon collecting sensor data, the data are then delivered to the information gathering phase as input. The preprocessing layer employs various information cleanup procedures and verification methods to eliminate inconsistencies in the individual data;
- A deep extreme learning machine (DELM) is applied for the dataset training process. The DELM carries the advantage of extreme learning as well as deep learning methodologies. The overall system process is illustrated in Figure 1. First, the data processing layer incorporates the input values; they travel to the neural network, where the framework has been trained to validate the dataset. Then, the DELM framework will train on data and export the trained model to the Cloud and send feedback to the validation phase for evaluation. The purpose of exporting the trained model to the cloud is to access it from anywhere.

If the patient is found to be infected in the validation phase, it sends the ACDA information that the patient is infected. The information of the infected patient is stored in a local database to update the dataset. If the dataset instance is greater than the given threshold point (e.g., 1000 new instances), then the entire collection of data will be exported to the WHO repository to retrain the model again for improving prediction accuracy; the more data we have, the more precision will be obtained.

### 2.2. Dataset

Table 1 contains a detailed description of the dataset used for COVID-19 detection. Headaches, flu, fever, cough, chest pain, shortness of breath, tiredness, nasal congestion, diarrhea, breathing difficulty, and pneumonia are all included in the dataset. The aforementioned characteristics are used to evaluate COVID-19 detection.

### 2.3. Architecture

The DELM architecture employs a variable number of hidden layers, many hidden neurons, and various activation functions to achieve the optimal framework for COVID-19 detection, as shown in Figure 1. Information gathering, pre-processing, and implementation are the three phases of the proposed architecture. The application layer includes two modes of evaluation, the second of which examines the outcomes of the current model’s predictions. Sensor data is initially collected for exploratory studies. Following the collection of sensor data, a layer for distributed data capture was applied. Then, in the pre-processing layer, various information cleanup processes and verification approaches eliminates anomalies from the individual data. Finally, the Deep Extreme Learning Machine (DELM) framework is implemented in the application layer to detect and reveal COVID-19-related symptoms of a person in a vehicle.

This machine learning method can be used to predict problems with overall health, indicating issues for users’ well-being, issues for long-term transportation availability, and even energy problems, and for other purposes, such as traffic monitoring [28,29,30]. However, because artificial neural architectures necessitate a wide range of measurement values and lengthy learning cycles, their effectiveness in producing correct results will outweigh the entire training cycle [31]. Therefore, the issues mentioned above are mitigated in the proposed algorithm. Huang [32] defines the definition of an extreme learning machine. Learning occurs in only one way in a feed-forward neural network. Nevertheless, we also apply the back-propagation strategy, which includes adjusting the network’s weights based on the input in order to achieve the highest accuracy while incurring the smallest mistake. To import a network that has been qualified, weights are set to equal real data, so they remain constant throughout the validation period. Once the model was prepared, we then sent it to the cloud for online use. To ensure consistency and accuracy, we then used the trained method on the cloud to generate expanded data sets, then validated it on the expanded data. The mean square error (MSE) is monitored at the assessment level to enhance COVID-19 prediction.

Using the DELM approach, we attempt to expand on the concept to obtain general or high-level results. Assume we have several Feedforward Neural Networks with a dataset of the number of layers with *n* neurons, each of which has the following hidden layers with ‘n’ inputs ($e_{i}$, $f_{i}$) in which $e_{i}\;\in \;S_{d}$ and $f_{i}\;\in \;S_{c}$. The outcome of these multiple hidden Layer Feedforward Neural network can be represented as [28]:

(1) $$\sum_{j=1}^{n}\gamma_{j}L\;\left(z_{j}e_{i}+a_{j}\right),i\in \left[1,N\right],$$

Here $z_{j}$ and $a_{j}$ denotes as acquiring parameters, $\gamma_{j}$ denotes as output of nodes weight j and L: S → S is the activation function [29].

(2) $$\sum_{j=1}^{n}\gamma_{j}L\left(z_{j}e_{i}+a_{j}\right)=f_{i},i\in \left[1,N\right]$$

which can be represented as

(3) $$Q_{\gamma }=F,$$

where

(4) $$Q=\left[\begin{array}{c} L\left(z_{1}e_{1}+a_{n}\right)\\ \begin{array}{c} \vdots \\ \vdots \end{array}\\ L\left(z_{1}e_{N}+a_{n}\right) \end{array}\ldots \;\begin{array}{c} L\left(z_{n}e_{1}+a_{n}\right)\\ \begin{array}{c} \vdots \\ \vdots \end{array}\\ L\left(z_{n}e_{N}+a_{n}\right) \end{array}\right]$$

and

(5) $$\gamma ={\left(\gamma_{1}^{T}\ldots \;\gamma_{n}^{T}\right)}^{T},\;F={\left(f_{1}^{T}\ldots \;f_{N}^{T}\right)}^{T}$$

Whenever the number of visible neurons exceeds the frequency of hidden neurons, the impact weight is computed by using the procedure as follows [29]:

(6) $$\gamma =Q^{∤}F$$

$Q^{∤}$ is the inverse matrix.

The backpropagation algorithm has weight configurations, feed-forward propagation, propagation of errors, and backward error distribution, in progression. An activation function like g(x) =  sigmoid is present in the hidden layer on each neuron. This assists in the design of the sigmoid function, which is then used to design the deep extreme learning machine network [28]:

(7) $$E=\frac{1}{2}\sum_{j}\left(\;sj-wp_{j}\right)2$$

where $s_{j}$ and $wp_{j}$ denotes the anticipated output and intended output, respectively. Equation (7) defines a backpropagation anomaly, which can be calculated by separating the square number of the desired outcome by two. The weight amendment is needed to account for the typical mistake. The output layer’s weight transition levels are denoted in Equation (8) [29].

(8) $$\Delta H_{i,j}^{l=6}\propto -\frac{\partial R}{\partial H^{l=6}}$$

 i=1,2,3…10 (no.of neurons)

and

j= Layer of Output Value

(9) $$\Delta H_{i,j}^{l=6}=-\mathrm{const}\frac{\partial R}{\partial H^{l=6}}$$

Write Equation (9) through the chain rule technique [29]:

(10) $$\Delta H_{i,j}^{l=6}=-\mathrm{const}\frac{\partial R}{\partial wp_{j}^{l}}\times \frac{\partial wp_{j}^{l}}{\partial NhH_{j}^{l}}\times \frac{\partial NhH_{j}^{l}}{\partial H_{i,j}^{l}}$$

The change in weight assessment can be obtained by replacing Equation (9) values as shown in Equation (10) [30].

(11a) $$\Delta H_{i,j}^{l=6}=\mathrm{const}\left(sj-wp_{j}\right)\times (wp_{j}^{l}\left(1-wp_{j}^{l}\right)\times wp_{j}^{l}$$

(11b) $$\Delta H_{i,j}^{l=6}={\mathrm{const}}_{j}wp_{j}^{l}$$

This is additionally complicated since, by weight relation, this contributes to miscalculations on every node [30].

From $H_{6}$ to $H_{1}\;$ or $H_{n}$

where n=5,4,3,2,1

(12a) $$\Delta H_{i,n}^{l}\propto -\left[\underset{j}{\sum }\;\frac{\partial R}{\partial wp_{j}^{l}}\times \frac{\partial wp_{j}^{l}}{\partial NhH_{j}^{l}}\times \frac{\partial NhH_{j}^{l}}{\partial wp_{n}^{l}}\;\right]\times \frac{\partial wp_{n}^{l}}{\partial NhH_{n}^{l}}\times \frac{\partial NhH_{n}^{l}}{\partial H_{i,n}^{l}}$$

(12b) $$\Delta H_{i,n}^{l}=R\left[\underset{j}{\sum }{϶}_{j}(H_{n,j}^{l})\right]\times wp_{n}^{l}\left(1-wp_{n}^{l}\right)\times Z_{i,\;n}$$

(12c) $$\Delta H_{i,n}^{l}={R϶}_{n}\;Z_{i,\;n}$$

where

(12d) $${϶}_{n}=\left[\underset{j}{\sum }{϶}_{j}(H_{n,j}^{l})\right]\times wp_{n}^{l}\left(1-wp_{n}^{l}\right)$$

The mechanism is to enhance the weights described and bias among output and the hidden layer illustrated in Equation (12e) [30].

(12e) $$\;H_{i,j}^{l=6}\left(t\right)=H_{i,j}^{l=6}\left(t\right)+\lambda \Delta H_{i,j}^{l=6}$$

Equation (13) demonstrates how weights are modified and how biases among the hidden layer and inputs exist [30].

(13) $$H_{i,n}^{l}\left(t\right)=H_{i,n}^{l}\left(t+1\right)+\lambda \Delta H_{i,j}^{l}$$

## 3. Simulation Results and Performance Evaluation

In this article, a DELM-based framework is implemented to the dataset. The information is randomly dispersed throughout 70% of the course (383 data samples), 30% of the dataset is utilized for validation (164 data samples). In this research, we assess the DELM to predict the system’s performance accurately. We use a variety of statistical methods to quantify the performance of the proposed scheme in Equations (14) and (15). The suggested DELM’s effectiveness is evaluated in this study.

(14) Miss rate=∑x=03(OxTj≠x)∑k=03(Tk)

where, *j* = 0, 1, 2, 3

(15) Accuracy=∑x=03(OxTx)∑x=03(Tx)

In Equations (14) and (15), O denotes the projecting output value of VCDS, and T denotes the actual output value. OxTx symbolizes that projecting and actual output values are similar. Likewise, OxTj≠x symbolizes error, where predictive and actual output value varies.

Table 2 shows the proposed VCDS for the prediction of COVID-19 during the training phase. Overall, a random 383 samples are employed during training, and these are then categorized into 70, 83, 27 and 203 records of Check-up required, Mild, Moderate, and Severe probability of COVID samples. As a result, it is indicated that 64 records of the check-up required class mean in which no COVID-19 is identified are precisely predicted, and 06 samples are incorrectly predicted as Mild COVID-19 positive. Similarly, in the case of Mild, Moderate, and Severe classes, 78, 15 and 203 samples are correctly identified, respectively.

Table 3 shows the proposed VCDS for the prediction of COVID-19 during the validation level. Overall, random samples of 148 are employed through validation that is categorized into 27, 36, 12 and 89 records of Check-up required, Mild, Moderate, and Severe probability of COVID samples. It is indicated that 24 records with a Check-up required class mean in which no COVID-19 identified are precisely predicted, and 03 samples are incorrectly predicted as Mild COVID-19 positive. Similarly, in the case of Mild, Moderate, and Severe classes, 32, 06 and 86 samples are correctly identified, respectively.

Table 4 displays the planned VCDS outputs in terms of precision and error rate for the training and validation procedure. It also observed that the proposed VCDS framework model achieves 92.69% accuracy with a 7.31% miss rate during the validation phase. And through validation, the proposed VCDS system model projected 90.24% accuracy with a 9.76% miss rate.

## 4. Discussion

In this article, a feature dataset was produced through a series of data preparation steps and was clinically demonstrated to be helpful in disease prediction. This dataset may be useful for detecting severe and moderate COVID-19 patients. In addition, utilizing the feature dataset, the DELM model properly identified the severe and moderate COVID-19 patients in this study. The total accuracy of the DELM model utilized was more than 92.69%. The findings in this publication have a strong predictive value for healthcare practitioners in recognizing COVID-19-infected individuals upon admission, both severely and mildly. The most effective supervised ML models in the paper may also be utilized as an integrated approach to mitigate negative aspects in healthcare units such as excessive workload and poor service quality.

## 5. Conclusions

It is critical to predict COVID-19 patients early in order to prevent the disease from spreading to others. In this study, we proposed a deep extreme learning machine-based approach to detecting COVID-19 in a vehicle using coronavirus disease-related symptoms obtained from hospitals. The proposed COVID-19 detection model achieved a greater than 90% accuracy. This study shows how deep extreme learning machines can be used to accommodate COVID-19 in the early stages. COVID-19 has now become a threat to global health infrastructure, and many people have died as a result of this lethal pandemic. Due to an increase in the number of emergency patients, healthcare professional capacity is limited, and computer-controlled autonomous system diagnosis in Vehicles can save lives through early medical assessment and appropriate treatment. Through effective and efficient training, the VCDS framework demonstrates outstanding efficiency in categorizing COVID-19 detection. We anticipate that the proposed framework will significantly improve the speed and precision of COVID-19 diagnostic cases. It could be useful in a disease outbreak where the epidemic risk and the need for prevention measures do not match the available resources. More appropriate, refined, and richer datasets will help the system’s learning rate to mature.

## Figures and Tables

**Table 1:** Dataset Description on VRS-4 Scale.

Headache		Flu		Fever	
No Pain	0	No Flu	0	No Fever	0
Mild Pain	1	Mild Flu	1	Mild Fever	1
Moderate Pain	2	Moderate Flu	2	Moderate Fever	2
Severe Pain	3	Severe Flu	3	Severe Fever	3
**Cough**		**Chest Pain**		**Breath Shortage**	
No Cough	0	No Pain	0	No shortage	0
Mild Cough	1	Mild Flu Pain	1	Mild shortage	1
Moderate Cough	2	Moderate Flu Pain	2	Moderate shortage	2
Severe Cough	3	Severe Flu Pain	3	Severe shortage	3
**Tiredness**		**Fatigue**		**Diarrhea**	
No tiredness	0	No fatigue	0	No diarrhea	0
Mild tiredness	1	Mild fatigue	1	Mild Diarrhea	1
Moderate tiredness	2	Moderate fatigue	2	Moderate Diarrhea	2
Severe tiredness	3	Severe fatigue	3	Severe Diarrhea	3
**Joint Pain**		**Travel History**		**Pneumonia**	
No Joint Pain	0	No travel history	0	No pneumonia	0
Mild Joint Pain	1	Mild travel history	1	Mild pneumonia	1
Moderate Joint Pain	2	Moderate travel history	2	Moderate pneumonia	2
Severe Joint Pain	3	Severe travel history	3	Severe pneumonia	3
**Close Contact with** **Suspected Person**		**Infected Family Member**		**COVID-19**	
No contact	0	None infected	0	Check-up Required	0
Mild contact	1	Mildly infected	1	Mild probability	1
Moderate contact	2	Moderately infected	2	Moderate probability	2
Severe contact	3	Severely infected	3	Severe probability	3

**Table 2:** Training of the suggested Vehicle-based COVID-19 Detection System (VCDS) during the prediction of COVID-19.

Proposed Vehicle-Based COVID-19 Detection System (70% Dataset for Training)					
Total No of records (N = 383)		Outcome (O_0_, O_1_, O_2_, O_3_)			
	Expected Output (T_0_, T_1_, T_2_, T_3_)	O_0_	O_1_	O_2_	O_3_
Input	T_0_ = 70 Check-up Required	64	6	0	0
	T_1_ = 83 Mild	3	78	2	0
	T_2_ = 27 Moderate	0	5	15	7
	T_3_ = 203 Severe	0	0	5	203

**Table 3:** Validation of the proposed Vehicle-based COVID-19 Detection System (VCDS) during the prediction of COVID-19.

Proposed Vehicle-Based COVID-19 Detection System (30% Dataset for Validation)					
Total No of records (N = 164)		Outcome (O_0_, O_1_, O_2_, O_3_)			
	Expected Output (T_0_, T_1_, T_2_, T_3_)	O_0_	O_1_	O_2_	O_3_
Input	T_0_ = 27 Check-up Required	24	3	0	0
	T_1_ = 36 Mild	3	32	1	0
	T_2_ = 12 Moderate	0	2	6	4
	T_3_ = 89 Severe	0	0	3	86

**Table 4:** Performance Evaluation of the suggested Vehicle-based COVID-19 Detection System (VCDS) in Validation & Training.

	Accuracy	Miss Rate
Training	92.69%	7.31%
Validation	90.24%	9.76%

## Data Availability

The simulation files/data used to support the findings of this study are available from the corresponding author upon request.
